# Thoracoscopic subtotal esophagectomy via a right thoracic cavity approach to treat an intractable fistula after 20 months from onset of an idiopathic esophageal rupture: A case report

**DOI:** 10.1111/ases.12736

**Published:** 2019-07-22

**Authors:** Takeharu Imai, Yoshihiro Tanaka, Takahito Adachi, Tomonari Suetsugu, Masahiro Fukada, Toshiyuki Tanahashi, Satoshi Matsui, Hisashi Imai, Takazumi Kato, Nobuhisa Matsuhashi, Takao Takahashi, Kazuya Yamaguchi, Takashi Shiroko, Kazuhiro Yoshida

**Affiliations:** ^1^ Department of Surgical Oncology, Graduate School of Medicine Gifu University Gifu Japan; ^2^ Department of Surgery Takayama Red Cross Hospital Takayama Japan

**Keywords:** idiopathic esophageal rupture, intractable fistula, thoracoscopic subtotal esophagectomy

## Abstract

An intractable fistula caused by idiopathic esophageal rupture is a rare but severe condition. In the present case, a 69‐year‐old man had been treated conservatively at another hospital for esophageal rupture but had developed an abscess in the left thoracic cavity due to an intractable fistula at the rupture site. He was referred to our hospital for treatment 19 months after the esophageal rupture. On admission, the intractable fistula was found to be continuous with an abscess in the left thoracic cavity. Preoperative continuous enteral nutrition was administered to improve the patient's nutritional status, and drainage was performed to reduce the size of the abscess. Then, to minimize the invasion of the intractable fistula, thoracoscopic subtotal esophagectomy was performed via a right thoracic cavity approach 20 months after the esophageal rupture. Preoperative management and thoracoscopic surgery via an opposite chest cavity approach was found to be safe and feasible for the intractable fistula caused by idiopathic esophageal rupture.

## 1 INTRODUCTION

Idiopathic esophageal rupture is a severe condition, and the reported mortality rate is 13.3%.^1^ The perforation also sometimes heals after drainage, but in some cases, a fistula may form, as was the case in the patient reported here.^2^ In the present case, we carried out surgery 20 months after the occurrence of idiopathic esophageal rupture. Esophageal rupture into the left thoracic cavity is normally treated with an approach via the left thoracic cavity.^3^, ^4^ However, our surgical approach chosen was to perform thoracoscopic surgery via the right thoracic cavity. Our successful treatment strategy consisted of reducing the size of the abscess by continuous irrigation and suction drainage and improving the patient's nutritional status by continuous enteral nutrition, followed by thoracoscopic surgery. There has been no previous case report of surgery to treat an intractable fistula due to idiopathic esophageal rupture after a long period of time has elapsed. This is a valuable case study of successful thoracoscopic surgery.

## 2 CASE PRESENTATION

A 69‐year‐old man developed idiopathic esophageal rupture, which was treated conservatively at another hospital through fasting, a nasogastric tube, and left thoracic cavity drainage. However, after 5 months, an intractable fistula and an abscess in the left thoracic cavity developed. An esophageal stent was inserted at the fistula site 11 months after the idiopathic esophageal rupture occurred; however, it did not seal the fistula. After 12 months, he underwent abscess drainage by left thoracotomy and jejunostomy. He had received enteral nutrition intermittently during the daytime. However, endoscopy confirmed enlargement of the fistula after 17 months (Figure S1). Because the patient continued to develop recurrent fevers, he was referred to our hospital for treatment 19 months after the esophageal rupture.

**Figure 1:**
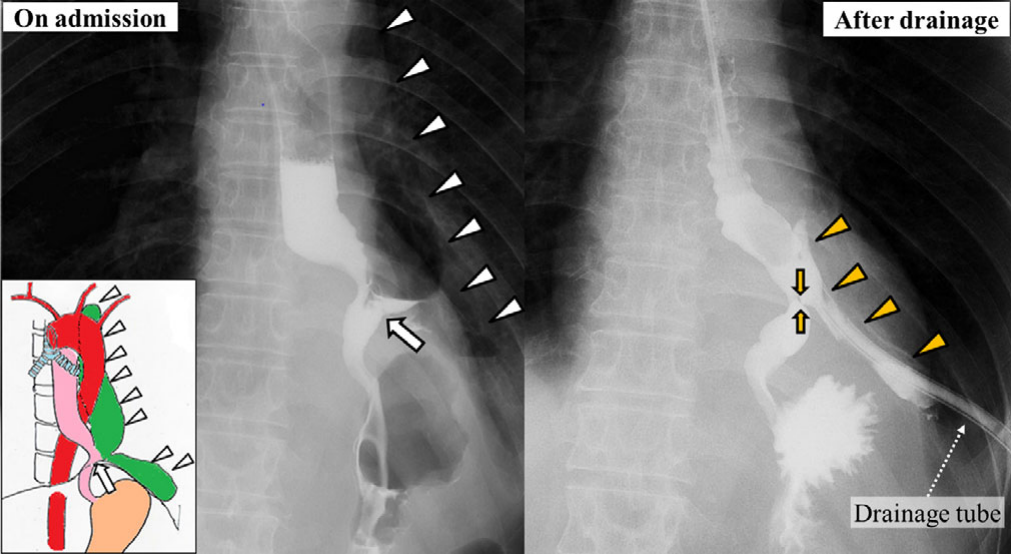
Gastrografin upper gastrointestinal contrast radiography on admission and after preoperative abscess drainage. On admission, a 2‐cm diameter fistula (white arrow) was detected in the left wall of the thoracic lower esophagus. The fistula was continuous with an abscess in the left thoracic cavity (white arrowheads). After preoperative abscess drainage, gastrografin radiography via the preoperatively inserted gastric tube and left abscess drain revealed that the fistula (orange arrows) and abscess (orange arrowheads) had both decreased in size

**Figure 2:**
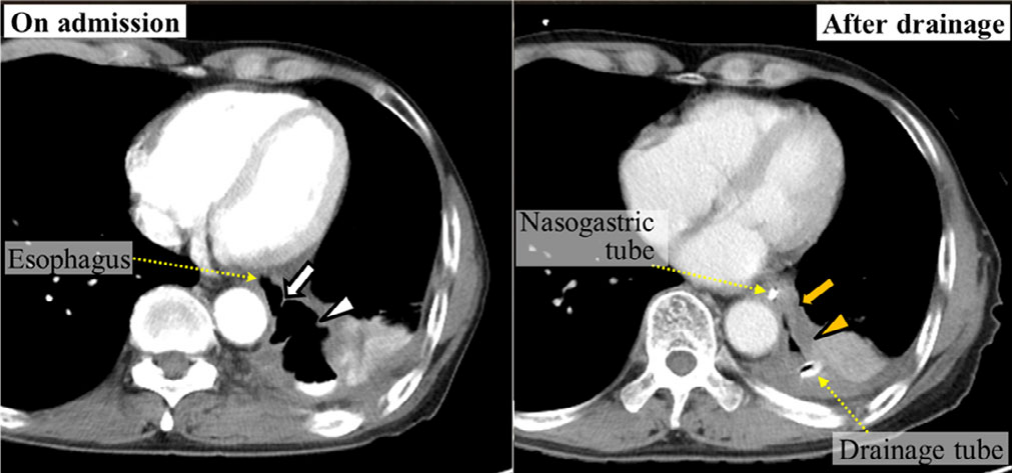
Contrast‐enhanced computed tomography on admission and after preoperative abscess drainage. On admission, the fistula in the left wall of the thoracic lower esophagus (white arrow) was continuous with an abscess in the left thoracic cavity (white arrowheads). After preoperative abscess drainage, the fistula (orange arrow) and abscess cavity (orange arrowheads) had both decreased in size

On admission, the patient's weight was 47.7 kg, with a body mass index (BMI) of 17.3. Blood tests revealed serum prealbumin levels of 7 mg/dL and a prognostic nutritional index (PNI) score of 41.9. The white blood cell count was 7010/μL and C‐reactive protein level was 8.38 mg/dL. Gastrografin contrast radiography (UGI) revealed a 2‐cm diameter fistula in the left wall of the lower thoracic esophagus (Figure 1). According to contrast‐enhanced computed tomography (CT), an abscess extended within the left thoracic cavity from immediately above the diaphragm to the tracheal bifurcation (Figure 2). As the fistula area was in contact with the pericardium and the descending aorta, the possibility of adhesions was a matter of concern. A decision was made to perform subtotal esophagectomy via a right thoracic cavity approach at our hospital. Therefore, it was very important to preoperatively minimize the size of the left thoracic abscess. So an 18‐Fr double‐lumen trochar was inserted percutaneously into the left thoracic cavity, and continuous irrigation with physiological saline and suction drainage were carried out. In addition, given that the patient was malnourished, he was started on 24‐hours continuous enteral nutrition using a jejunostomy tube. After 1 month, the patient's weight had increased to 49.8 kg and his BMI had improved to 18.5. His serum prealbumin levels had improved to 26 mg/dL and PNI to 46.3. Gastrografin radiography and enhanced CT revealed that the abscess and fistula had both decreased in size (Figures 1 and 2).

**Figure 3:**
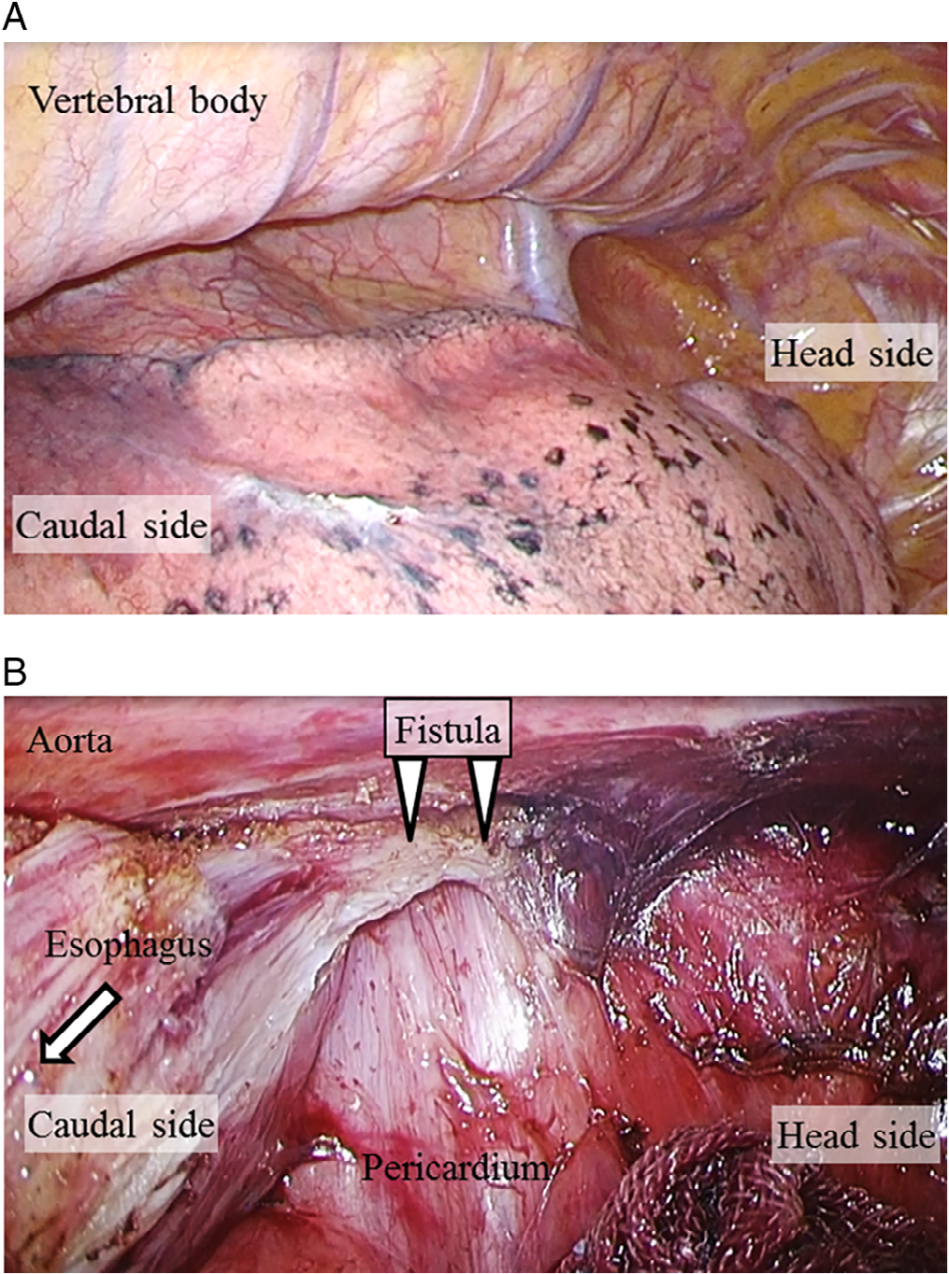
Intraoperative photographs. A, There were no adhesions in the right thoracic cavity. B, Mild adhesions were present around the fistula. The esophagus was placed under traction (arrow) during the approach to the fistula site (arrowheads)

Thoracoscopic subtotal esophagectomy via a right thoracic cavity approach with the patient in the left semi‐prone position was performed 20 months after the idiopathic esophageal rupture had occurred (Figure S2A). Four ports were placed in the right lateral side of the chest wall (Figure S2B). Artificial pneumothorax was induced with insufflation pressure of 10 mm Hg, and the right lung was deflated and anteriorly displaced. Intraoperative examination did not reveal any adhesions in the right thoracic cavity (Figure 3A). The esophagus was easily mobilized in the upper and middle mediastinum (Figure S2C,D). Because mild adhesions were present in the lower mediastinum, the esophagus was first transected caudad to the tracheal bifurcation and was then placed under traction during approach to the fistula site (Figure S2E). A cord‐like structure that was presumed to be the fistula was identified and resected (Figure 3B). The esophagus just above the diaphragm was displaced toward the left thoracic cavity, which appeared to be the effect of chronic inflammation due to perforation (Figure S2F). A trochar was inserted into the right thoracic cavity, and the drain in the left abscess was left in place. The abdominal esophagus was dissected transhiatally via an open abdominal approach with the patient in the supine position, and the specimen was removed. The esophagus was then reconstructed using the subtotal stomach via a retrosternal route.

Postoperative extubation was carried out in the operating room. The drain in the left thoracic cavity was removed on postoperative day (POD) 3. The patient started eating orally on POD 7 and was discharged on POD 13. Six months postoperatively, there has been no recurrence of the abscess in the left thoracic cavity.

## 3 DISCUSSION

Recently, a number of case reports have been published on successful conservative therapy using esophageal stents.^2^, ^5^ However, surgery is still required in cases in which the perforation cannot be covered by insertion of a stent.^2^ Factors reported for an unsuccessful stent placement were early stent migration, undersizing the stent in length or diameter, and improper positioning.^2^ In the present case, an esophageal stent was inserted caudal to the fistula, which did not seal the fistula. Given the lower chest esophagus was anatomically bent from chronic inflammation due to perforation, proper insertion of the stent might have been difficult (Figure 1).

One study found that fistulas formed as a result of esophageal rupture in 15.9% of cases.^1^ In some cases, esophageal stent insertion may also result in the formation of a new fistula, and surgery to close the rupture site may also cause fistula formation, necessitating further surgery.^6^, ^7^

In the present case, as significant time had elapsed since the abscess had formed, possible adhesions in the left thoracic cavity were a matter of concern. Because we wanted to avoid creating any communication between the abscess and the left thoracic cavity, the right thoracic cavity approach was chosen with the intention of minimizing invasion. The absence of adhesions in the right thoracic cavity was confirmed by intraoperative thoracoscopy via the camera port. This also enabled the fistula site to be thoroughly dissected under a magnified view, making the most of the advantages of endoscopic surgery.

For thoracoscopic surgery via the right thoracic cavity to be successful, it was very important to preoperatively minimize the size of the left thoracic abscess and to improve the patient's nutritional status. Thus, we performed continuous percutaneous irrigation and drainage for 1 month preoperatively to minimize the size of the abscess in the left thoracic cavity. Continuous irrigation and drainage improved the elevated inflammatory reaction and reduced the size of the abscess as intended. As a result, it was possible to remove the drain in the abscess in the left thoracic cavity after only 3 days following surgery.

We also improved the patient's nutritional status during preoperative management. In general, the gastrointestinal tract can be safely resected and anastomosed in patients with a PNI score of 45 points or higher.^8^ However, both the patient's PNI score and serum prealbumin level were low on admission. Before his admission to our department, the patient had only received enteral nutrition intermittently during the day. Protein ingested before sleeping is more easily digested and absorbed overnight.^9^ After 1 month of 24 hours continuous enteral nutrition, his nutritional status was improved. We therefore considered that providing enteral nutrition overnight would be effective.

In conclusion, when treating an intractable fistula formation that has developed over time because of an idiopathic esophageal rupture, thoracoscopic subtotal esophagectomy via a right thoracic cavity approach after preoperative management might be an effective method of treatment.

## AUTHOR CONTRIBUTIONS

TI, YT, TA TS and KY managed the patient. TI and YT wrote the manuscript and provided the original pictures. All the other authors reviewed the manuscript. All authors approved the final manuscript.

## Supporting information


**Figure S1.** Endoscopic view 17 months from onset of an idiopathic esophageal rupture. The fistula (arrow) and esophagogastric junction (arrowhead) are shown.

**Figure S2.** Intraoperative photographs. A, Diagram of the right thoracic cavity approach. The arrow indicates the fistula. B, Diagram of the left semi‐prone position and the trocar sites, showing 12‐mm ports at the third and seventh intercostal spaces on the midaxillary line; a 12‐mm port for thoracoscopy at the ninth intercostal space on the posterior axillary line; and a 5‐mm port at the fifth intercostal space on the midaxillary line. C, In the right upper mediastinum, there were no adhesions and removal around the esophagus was easy. D, In the right middle mediastinum, there were no adhesions on the cranial side of the tracheal bifurcation (arrows). E, Caudal to the tracheal bifurcation, mild adhesions were observed around the esophagus. F, There were mild adhesions in the right lower mediastinum around the fistula and the crura of the diaphragm. The esophagus just above the diaphragm was displaced toward the left thoracic cavity.
