# Supplementary figures and images for: Thoracoscopic subtotal esophagectomy via a right thoracic cavity approach to treat an intractable fistula after 20 months from onset of an idiopathic esophageal rupture: A case report
Source: Asian J Endosc Surg. 2019 Jul 22;13(3):402–5. doi: 10.1111/ases.12736 (PMC7379975; doi:10.1111/ases.12736)

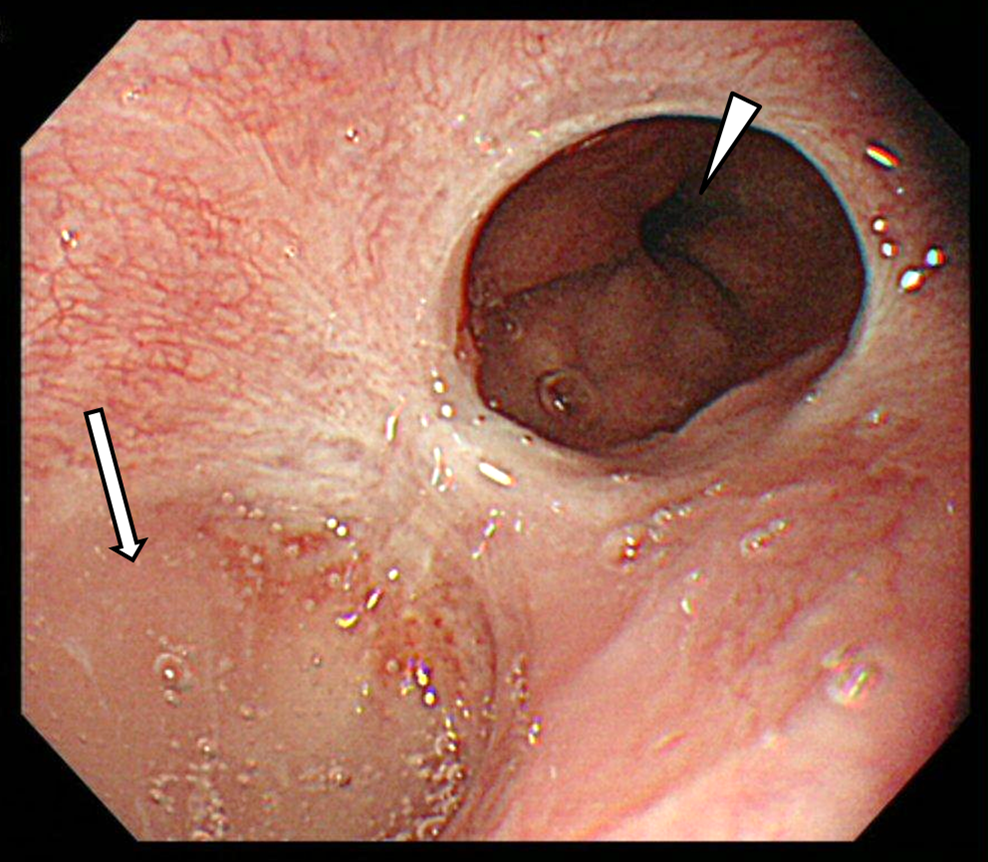

Supplement: Supplementary file 1 — Figure S1. Endoscopic view 17 months from onset of an idiopathic esophageal rupture. The fistula (arrow) and esophagogastric junction (arrowhead) are shown. [file ASES-13-402-s001.tif]

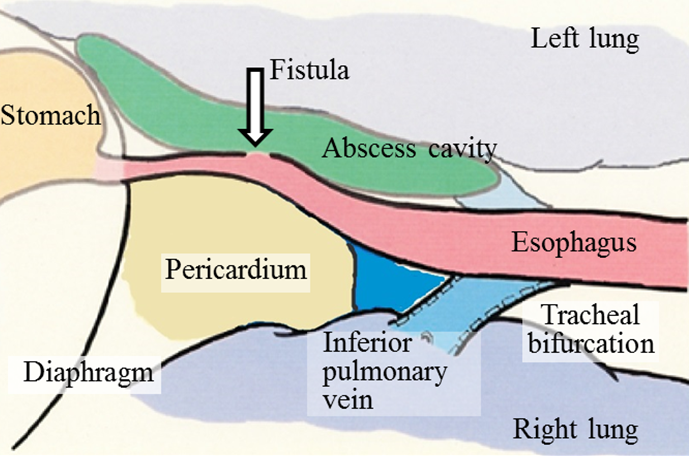

Supplement: Supplementary file 2 — Figure S2. Intraoperative photographs. A, Diagram of the right thoracic cavity approach. The arrow indicates the fistula. B, Diagram of the left semi‐prone position and the trocar sites, showing 12‐mm ports at the third and seventh intercostal spaces on the midaxillary line; a 12‐mm port for thoracoscopy at the ninth intercostal space on the posterior axillary line; and a 5‐mm port at the fifth intercostal space on the midaxillary line. C, In the right upper mediastinum, there were no adhesions and removal around the esophagus was easy. D, In the right middle mediastinum, there were no adhesions on the cranial side of the tracheal bifurcation (arrows). E, Caudal to the tracheal bifurcation, mild adhesions were observed around the esophagus. F, There were mild adhesions in the right lower mediastinum around the fistula and the crura of the diaphragm. The esophagus just above the diaphragm was displaced toward the left thoracic cavity. [file ASES-13-402-s002.zip › ASES_12736_Supplementary Figure S2A(300dpi).tif]

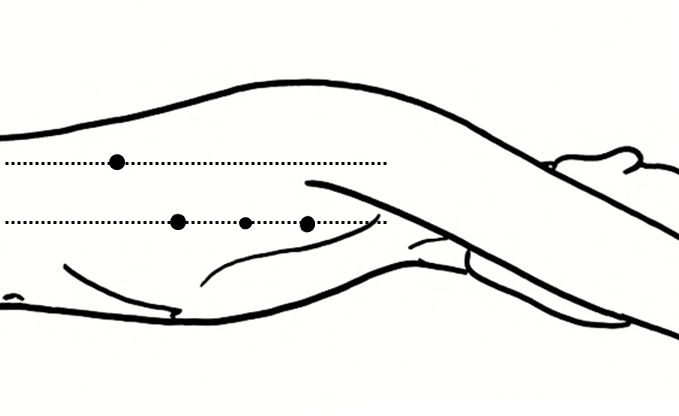

Supplement: Supplementary file 2 — Figure S2. Intraoperative photographs. A, Diagram of the right thoracic cavity approach. The arrow indicates the fistula. B, Diagram of the left semi‐prone position and the trocar sites, showing 12‐mm ports at the third and seventh intercostal spaces on the midaxillary line; a 12‐mm port for thoracoscopy at the ninth intercostal space on the posterior axillary line; and a 5‐mm port at the fifth intercostal space on the midaxillary line. C, In the right upper mediastinum, there were no adhesions and removal around the esophagus was easy. D, In the right middle mediastinum, there were no adhesions on the cranial side of the tracheal bifurcation (arrows). E, Caudal to the tracheal bifurcation, mild adhesions were observed around the esophagus. F, There were mild adhesions in the right lower mediastinum around the fistula and the crura of the diaphragm. The esophagus just above the diaphragm was displaced toward the left thoracic cavity. [file ASES-13-402-s002.zip › ASES_12736_Supplementary Figure S2B(300dpi).tif]

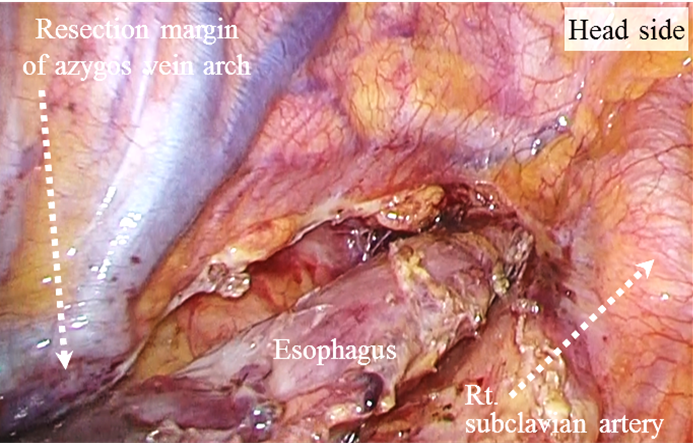

Supplement: Supplementary file 2 — Figure S2. Intraoperative photographs. A, Diagram of the right thoracic cavity approach. The arrow indicates the fistula. B, Diagram of the left semi‐prone position and the trocar sites, showing 12‐mm ports at the third and seventh intercostal spaces on the midaxillary line; a 12‐mm port for thoracoscopy at the ninth intercostal space on the posterior axillary line; and a 5‐mm port at the fifth intercostal space on the midaxillary line. C, In the right upper mediastinum, there were no adhesions and removal around the esophagus was easy. D, In the right middle mediastinum, there were no adhesions on the cranial side of the tracheal bifurcation (arrows). E, Caudal to the tracheal bifurcation, mild adhesions were observed around the esophagus. F, There were mild adhesions in the right lower mediastinum around the fistula and the crura of the diaphragm. The esophagus just above the diaphragm was displaced toward the left thoracic cavity. [file ASES-13-402-s002.zip › ASES_12736_Supplementary Figure S2C(300dpi).tif]

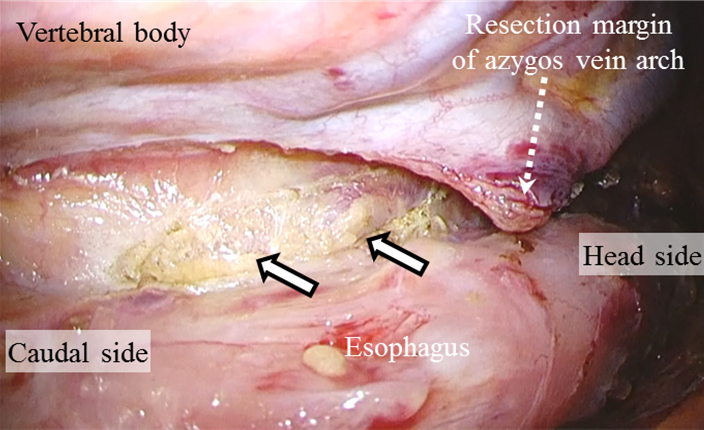

Supplement: Supplementary file 2 — Figure S2. Intraoperative photographs. A, Diagram of the right thoracic cavity approach. The arrow indicates the fistula. B, Diagram of the left semi‐prone position and the trocar sites, showing 12‐mm ports at the third and seventh intercostal spaces on the midaxillary line; a 12‐mm port for thoracoscopy at the ninth intercostal space on the posterior axillary line; and a 5‐mm port at the fifth intercostal space on the midaxillary line. C, In the right upper mediastinum, there were no adhesions and removal around the esophagus was easy. D, In the right middle mediastinum, there were no adhesions on the cranial side of the tracheal bifurcation (arrows). E, Caudal to the tracheal bifurcation, mild adhesions were observed around the esophagus. F, There were mild adhesions in the right lower mediastinum around the fistula and the crura of the diaphragm. The esophagus just above the diaphragm was displaced toward the left thoracic cavity. [file ASES-13-402-s002.zip › ASES_12736_Supplementary Figure S2D(300dpi).tif]

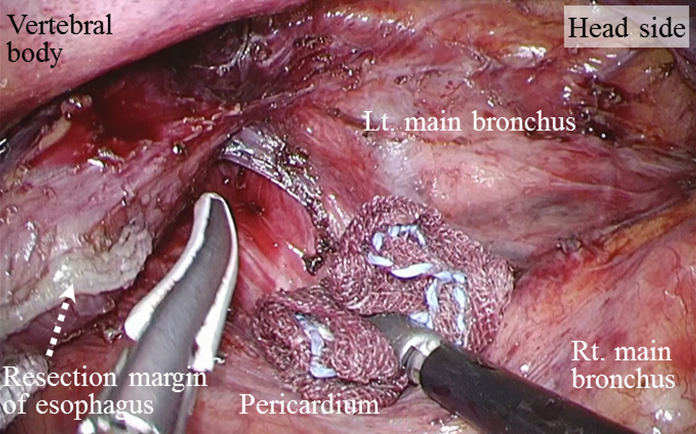

Supplement: Supplementary file 2 — Figure S2. Intraoperative photographs. A, Diagram of the right thoracic cavity approach. The arrow indicates the fistula. B, Diagram of the left semi‐prone position and the trocar sites, showing 12‐mm ports at the third and seventh intercostal spaces on the midaxillary line; a 12‐mm port for thoracoscopy at the ninth intercostal space on the posterior axillary line; and a 5‐mm port at the fifth intercostal space on the midaxillary line. C, In the right upper mediastinum, there were no adhesions and removal around the esophagus was easy. D, In the right middle mediastinum, there were no adhesions on the cranial side of the tracheal bifurcation (arrows). E, Caudal to the tracheal bifurcation, mild adhesions were observed around the esophagus. F, There were mild adhesions in the right lower mediastinum around the fistula and the crura of the diaphragm. The esophagus just above the diaphragm was displaced toward the left thoracic cavity. [file ASES-13-402-s002.zip › ASES_12736_Supplementary Figure S2E(300dpi).tif]

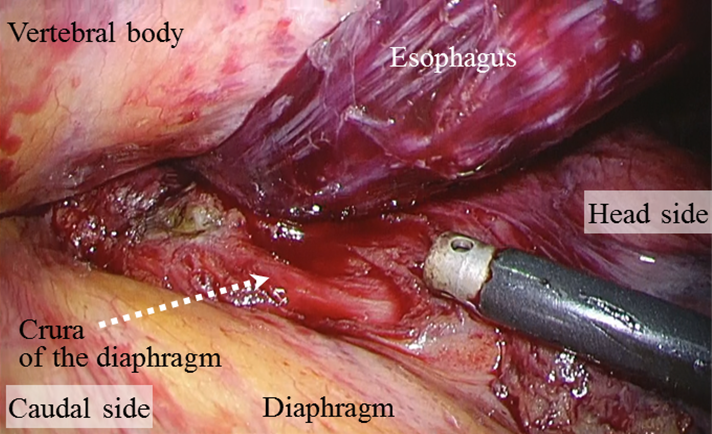

Supplement: Supplementary file 2 — Figure S2. Intraoperative photographs. A, Diagram of the right thoracic cavity approach. The arrow indicates the fistula. B, Diagram of the left semi‐prone position and the trocar sites, showing 12‐mm ports at the third and seventh intercostal spaces on the midaxillary line; a 12‐mm port for thoracoscopy at the ninth intercostal space on the posterior axillary line; and a 5‐mm port at the fifth intercostal space on the midaxillary line. C, In the right upper mediastinum, there were no adhesions and removal around the esophagus was easy. D, In the right middle mediastinum, there were no adhesions on the cranial side of the tracheal bifurcation (arrows). E, Caudal to the tracheal bifurcation, mild adhesions were observed around the esophagus. F, There were mild adhesions in the right lower mediastinum around the fistula and the crura of the diaphragm. The esophagus just above the diaphragm was displaced toward the left thoracic cavity. [file ASES-13-402-s002.zip › ASES_12736_Supplementary Figure S2F(300dpi).tif]
